# Targeting T cells to treat atherosclerosis: odyssey from bench to bedside

**DOI:** 10.1093/ehjcvp/pvw001

**Published:** 2016-01-24

**Authors:** Jessica Bullenkamp, Sip Dinkla, Juan Carlos Kaski, Ingrid E. Dumitriu

**Affiliations:** Cardiovascular and Cell Sciences Research Institute, St George's, University of London, Cranmer Terrace, London SW17 0RE, UK

**Keywords:** Atherosclerosis, Immune response, Immunomodulation, Inflammation, T lymphocytes

## Abstract

More than 150 years from the initial description of inflammation in atherosclerotic plaques, randomized clinical trials to test anti-inflammatory therapies in atherosclerosis have recently been initiated. Lymphocytes and macrophages are main participants in the inflammatory response in atherosclerosis. T lymphocytes operate mainly by exerting strong influences on the function of many cells in the immune system and beyond, and co-ordinating their interactions. Importantly, T lymphocytes are not a homogenous population, but include several subsets with specialized functions that can either promote or suppress inflammation. The interactions between these T-lymphocyte subsets have critical consequences on the course and outcome of inflammation. The complexity of the inflammatory response in atherosclerosis poses significant challenges on translating experimental findings into clinical therapies and makes the journey from bench to bedside an arduous one. Here, we summarize recent advances on the role of CD4^+^ T cells in the inflammatory process in atherosclerosis and discuss potential therapies to modulate these lymphocytes that may provide future breakthroughs in the treatment of atherosclerosis.

## Introduction

Atherosclerosis was initially considered a disease exclusively caused by cholesterol deposition in the arterial wall. Significant evidence demonstrated that atherosclerotic plaques are not passive collections of lipids, but sites of active interactions between cells of the immune system and vascular cells that influence the fate of atheromas.^1^ As evidenced in several studies in animal models and patients, both innate and adaptive immune cells participate in this process and have significant effects on the initiation and progression of atherosclerotic lesions.^2,3^ Interactions between immune and vascular cells trigger a self-perpetuating inflammatory cycle that generates a chronic inflammatory milieu that promotes atherosclerotic plaque growth and rupture.^1^ The hypothesis that inflammation is a significant driving factor in atherosclerosis is now the focus of phase III clinical trials that test strategies to reduce inflammatory mediators.^4^ On-going phase III trials target the innate inflammatory cytokines interleukin-1β (IL-1β; targeted with Canakinumab, a human monoclonal anti-IL-1β antibody in the CANTOS trial), IL-6, and tumour necrosis factor-α (TNF-α; targeted with low-dose methotrexate in the CIRT trial). The immune response in atherosclerosis is multifaceted and, in addition to innate cells, adaptive immune responses have significant contributions. Indeed, adaptive immune cells such as T lymphocytes, B lymphocytes, and antibodies have been identified in atherosclerotic plaques and circulation of patients with atherosclerosis.^5^ Moreover, substantial evidence from animal studies clearly demonstrates that specialized subsets of T and B lymphocytes exert either protective or promoting effects on atherogenesis.^2^ These data suggest that more targeted approaches may be required to successfully retune the immune system and modulate the complex inflammatory response in atherosclerosis. In the following sections, we will summarize recent advances on the role of CD4^+^ T lymphocytes in atherosclerosis, focusing on two subsets [CD4^+^CD28^null^ and regulatory T (Treg) cells] that are at opposing poles of the inflammatory process that underlies atherosclerosis and harbour therapeutic potential.

## CD4^+^ T cells in atherosclerosis

T lymphocytes represent the second largest immune cell population in atherosclerotic plaques after macrophages. Both helper (CD4^+^) and cytotoxic (CD8^+^) T cells have been identified in atheromas. However, the inflammatory response in atherosclerosis is dominated by Th1 lymphocytes, the most abundant T-cell subset in plaques. Characteristically, Th1 cells are identified by the production of interferon-γ (IFN-γ), a cytokine that promotes atherosclerosis and plaque rupture through effects on several cells in atherosclerotic lesions.^1^ While the pro-atherogenic role of Th1 is well documented, the precise contribution of other CD4^+^ T-cell subsets (e.g. Th2 and Th17) remains debatable due to conflicting reports.^2^

## CD4^+^CD28^null^ T cells

**Figure 1:**
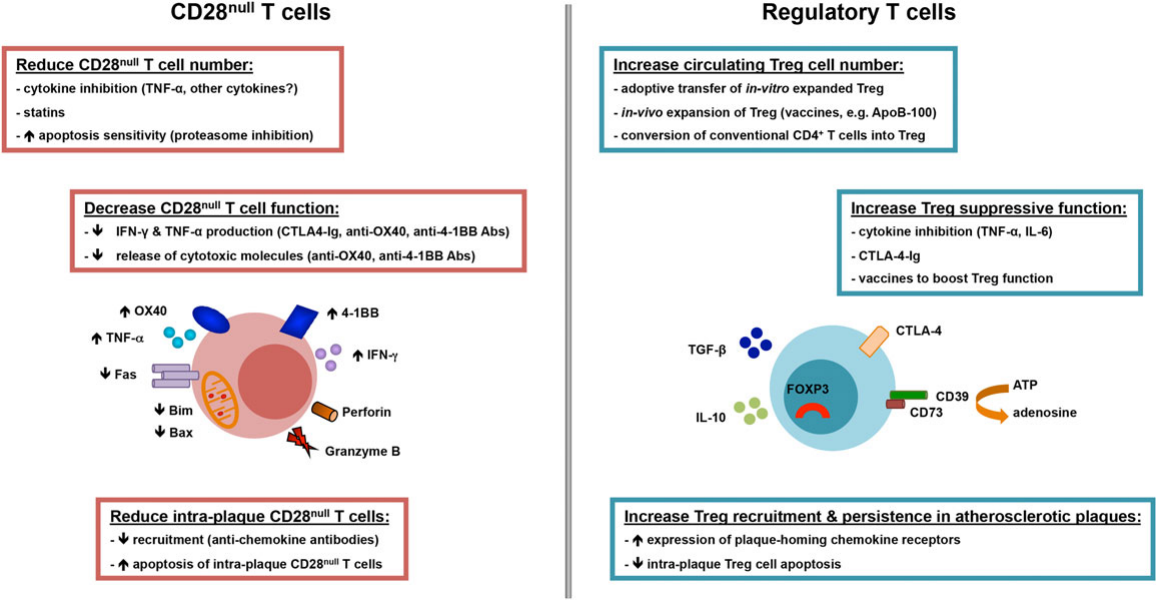
Overview of cardinal features and potential strategies to target CD28^null^ and regulatory T-cell lymphocytes in atherosclerosis. CD28^null^ T cells expand in the circulation and atherosclerotic plaques in patients with acute coronary syndrome. These cells produce high levels of inflammatory cytokines (interferon-γ and tumour necrosis factor-α) and express and release cytotoxic molecules (perforin and granzyme B). The production of cytokines and cytotoxic molecules is modulated by co-stimulatory receptors OX40 and 4-1BB, which are up-regulated on CD28^null^ T cells. Moreover, CD28^null^ T cells are resistant to apoptosis due to reduction in apoptotic molecules Fas, Bim, and Bax, which underlies the expansion of this cell subset in acute coronary syndrome patients. Several strategies have been proposed to reduce the number and/or function of CD28^null^ T cells such as tumour necrosis factor-α inhibition, statins, sensitization to apoptosis, and inhibition of co-stimulation (CTLA4-Ig, anti-OX40, and anti-4-1BB antibodies). Regulatory T cells have crucial roles in counteracting the actions of inflammatory T lymphocytes such as CD28^null^ T cells. Redundant mechanisms enable regulatory T cells to suppress inflammation and T-cell actions among which are the production of anti-inflammatory cytokines (interleukin-10 and transforming growth factor-β), CTLA-4-mediated suppression, and interference with metabolic pathways (conversion of ATP into adenosine, which has anti-inflammatory properties). On-going experimental and clinical work is testing various strategies to boost the number and/or the function of circulating or intraplaque regulatory T cells such as adoptive transfer of regulatory T cells expanded *in vitro*, vaccination protocols that induce expansion of naturally occurring regulatory T cells or conversion of conventional CD4^+^ T cells into regulatory T cells, and blockade of inflammatory cytokines (tumour necrosis factor-α and interleukin-6).

Most naive CD4^+^ T cells constitutively express the co-stimulatory receptor CD28, which delivers vital signals that sustain the proliferation and survival of T cells upon antigen recognition.^6^ A subset of CD4^+^ T cells that lack CD28—known as CD4^+^CD28^null^ (CD28^null^) T cells—has been identified and implicated in several chronic inflammatory diseases, including atherosclerosis.^7,8^ These cells share features with Th1 cells, but also diverge phenotypically and functionally from conventional CD4^+^CD28^+^ (CD28^+^) Th1 lymphocytes (*Figure 1*).^7,9^ Noteworthy, CD28^null^ T cells are more adept at secreting pro-inflammatory cytokines IFN-γ and TNF-α than conventional CD28^+^ T cells, both in the resting state and following activation.^10^ We have recently demonstrated that, in patients with acute coronary syndrome (ACS), CD28^null^ T-cell cytokine production is regulated by the alternative co-stimulatory receptors OX40 and 4-1BB^6^ that were significantly up-regulated on circulating CD28^null^ compared with CD28^+^ T cells.^10^ In addition, CD28^null^ T cells express and release cytotoxic molecules perforin and granzyme B.^10,11^ These molecules, usually restricted to cytotoxic CD8^+^ T cells and natural killer cells, have been suggested to enable CD28^null^ T cells to lyse endothelial cells *in vitro*.^12^ We found that, in ACS, OX40 and 4-1BB regulate not only the cytokine production, but also the release of perforin from CD28^null^ T cells.^10^ Another distinguishing feature from conventional CD28^+^ T lymphocytes is the reduced sensitivity of CD28^null^ T cells to apoptosis induction. In rheumatoid arthritis (RA), this has been attributed to an increase in anti-apoptotic proteins (e.g. Bcl-2),^13^ while in ACS we found a reduction in pro-apoptotic proteins (e.g. Fas, Bim, and Bax).^14^ Other characteristics of CD28^null^ T cells have been described elsewhere.^9^

Whereas circulating CD28^null^ T cells are nearly undetectable in healthy individuals, this subset expands in patients with ACS,^10,15^ chronic inflammatory disorders such as autoimmunity (extensively characterized in RA), chronic infections, and chronic or end-stage kidney disease.^9^ Acute coronary syndrome patients show significantly higher frequencies of circulating CD28^null^ T cells than healthy controls or stable angina (SA) patients.^10,15^ A 4-year follow-up study found that ACS patients with recurrent myocardial infarction had four-fold higher circulating CD28^null^ T-cell frequencies compared with patients with only one acute event and that the expansion of CD28^null^ T lymphocytes associated with ACS severity and poor outcome, indicating a pathogenic role for these cells.^16^ This is also supported by identification of CD28^null^ T cells not only in the peripheral circulation but also in atherosclerotic plaques, with preferential accumulation in unstable lesions.^17^ As CD28^null^ T cells secrete IFN-γ^10,18^ and lyse endothelial cells,^12^ these effector functions may promote local inflammation and trigger plaque destabilization.

Importantly, RA and other inflammatory diseases associate with increased ACS incidence and poorer outcome.^19^ It is tempting to speculate that CD28^null^ T cells are likely candidates for driving the accelerated atherosclerosis process observed in these disorders. Indeed, RA patients with high frequencies of CD28^null^ T lymphocytes have increased carotid artery intima-media thickness (IMT) and decreased flow-mediated vasodilatation than those in whom this subset does not expand.^20^ Similarly, the presence of CD28^null^ T cells in end-stage kidney disease patients has been associated with increased C-reactive protein and IMT.^21^ Moreover, CD28^null^ T-cell expansion in patients with type II diabetes correlates with ACS occurrence and poor outcome.^18^

The mechanisms involved in CD28^null^ T-cell expansion in atherosclerosis and inflammatory disorders remain poorly understood. It has been suggested that CD28 downregulation is antigen-driven.^17^ Several exogenous and endogenous antigens have been proposed including cytomegalovirus and heat shock proteins (HSPs).^22,23^ Strikingly, CD28^null^ T cells that react to oxidized LDL (ox-LDL), one of the antigens frequently implicated in atherosclerosis, have not been described.^23^ An alternative hypothesis implicates inflammatory cytokines in CD28^null^ T-cell expansion. CD28^+^ T-cell clones from RA patients downregulated CD28 transcription following TNF-α treatment *in vitro*.^24^ Conversely, infliximab triggered CD28 re-expression in cells from patients with RA or unstable angina (UA) *in vitro*;^25,26^ however, a different study found that etanercept and infliximab did not affect the percentage of circulating CD28^null^ T cells in RA patients.^27^ Another mechanism that may underlie CD28^null^ T-cell expansion is resistance to apoptosis, due to defects in molecules regulating apoptosis entry.^13,14^

It has been suggested that CD28^null^ T cells may link innate and adaptive immune responses. In line with this hypothesis, CD28^null^ T lymphocytes from patients with RA, psoriatic arthritis, or ankylosing spondylitis (AS) were found to express Toll-like receptors TLR4 and TLR2 (the latter identified only in CD28^null^ T cells in AS).^28^ The frequency of CD28^null^ T-cell-expressing TLRs varied, with a median of roughly 25% for CD28^null^TLR4^+^ T cells and <5% for CD28^null^TLR2^+^ T cells. *In vitro* treatment with TNF-α up-regulated TLR4 and TLR2 expression on CD28^null^ T cells from AS patients. Contrastingly, TNF-α neutralization in AS patients decreased expression of these TLRs on circulating CD28^null^ T cells analysed *ex vivo*.^28^ These receptors were functional as demonstrated by increased perforin expression in CD28^null^ T cells following stimulation with the TLR4 and TLR2 agonist lipopolysaccharide.^28^ Of note, both exogenous (microbial) and endogenous (e.g. HSP60) TLR agonists have been described in atherosclerotic plaques, and TLR2/4-mediated signals have been implicated in atherosclerosis.^29^ Whether TLR agonists contribute to CD28^null^ T-cell activation *in situ* in atherosclerotic lesions warrants further investigation. Overall, CD28^null^ T cells produce high levels of inflammatory cytokines, release cytotoxic molecules, and infiltrate atherosclerotic lesions, wherein these features may allow them to contribute to the on-going inflammatory response and plaque destabilization.

## Regulatory CD4^+^ T cells

The actions of pro-inflammatory T lymphocytes are normally restrained by Treg cells. This specialized subset has critical roles in immune homeostasis and preventing excessive immune responses.^30,31^ The most numerous and best-characterized are thymus-derived (naturally occurring) Treg (identified as CD4^+^CD25^high^CD127^low^FOXP3^+^ T cells), as opposed to peripherally derived (induced) Treg, which originate from naive conventional T cells.^31^ Regulatory T cells comprise around 5% of CD4^+^ T cells in the peripheral blood in humans, and are characterized by the expression of the Forkhead box P3 transcription factor (FOXP3), high CD25 levels, and low/no CD127 expression.^30,31^ Forkhead box P3 transcription factor is essential for Treg development and suppressive function.^32^ Regulatory T cells employ several mechanisms to suppress effector cells, among which are inhibitory cell–cell interactions, release of anti-inflammatory cytokines (IL-10 and transforming growth factor-β, TGF-β), and disruption of metabolic pathways (*Figure 1*).^30,31^ Regulatory T-cell impairment through numerical and/or functional defects has been implicated in autoimmune diseases including type I diabetes, systemic lupus erythematosus, RA, multiple sclerosis, and inflammatory bowel disease.^31^

Evidence from animal models and patients with atherosclerosis suggests an overall protective role for Treg. Depletion of Treg in Apoe^−/−^ or Ldlr^−/−^ murine models aggravated atherosclerosis, with Treg suggested to limit plaque inflammation and disease progression, although the mechanisms responsible remain poorly defined.^33–35^ Data on Treg in human atherosclerosis are scant and fraught with contradictory findings. Initial studies suggested that the percentage of circulating CD4^+^CD25^+^ Treg is reduced in ACS compared with SA patients and healthy individuals.^36,37^ Recent studies failed to identify a consistent correlation between the percentage of circulating CD4^+^CD25^high^CD127^low^ Treg and the severity of coronary artery disease,^38^ whereas other authors suggested that CD4^+^FOXP3^+^ Treg reduction associates with an increased risk for myocardial infarction.^39^ A likely explanation for these contradictory findings is the inconsistent use of accurate markers to identify Treg, particularly FOXP3, which remains the most specific marker for delineating Treg from other T cells in humans.^32^ A recent study quantified Treg by assessing the demethylation of a conserved non-coding sequence in the *FOXP3* locus (the Treg cell-specific demethylated region), a feature essential for Treg suppressive function.^30,31^ Regulatory T cells identified by this method were reduced in ACS patients compared with controls, and their reduction correlated with ACS severity.^40^ Even less information is available on the suppressive function of Treg in patients with atherosclerosis. A report published in 2006 suggested a reduced suppressive function of circulating CD4^+^CD25^high^ Treg in ACS patients,^36^ but the study was insufficiently powered and did not employ a robust suppression assay.

Compared with other inflammed tissues, relatively low levels of FOXP3^+^ Tregs were observed in human atherosclerotic plaques (0.5–5% of CD3^+^ T cells), which may explain persistent inflammation in these lesions.^41^ Moreover, fewer FOXP3^+^ Treg were present in vulnerable rather than stable plaques.^42^ Impaired Treg survival has been suggested to have a role in this process, and *in vitro* studies indicate that ox-LDL may trigger Treg apoptosis.^43^ Recent data in ACS patients suggest that circulating CD4^+^ T cells may have impaired ability to differentiate into Treg due to increased expression of protein tyrosine phosphatase PTPN22.^44^ A different study suggested that CD4^+^CD25^high^CD127^low^ Treg are enriched in coronary thrombi adjacent to culprit lesions compared with peripheral blood in ACS patients and that Treg from thrombi express a restricted repertoire of antigen receptors compared with circulating Treg.^45^ This suggests that circulating Treg may migrate into atherosclerotic lesions to control the inflammatory response, although further work is warranted to clarify the contribution of circulating and plaque-resident Treg in human atherosclerosis.

## Potential strategies to target CD28^null^ T cells

Several attempts have been made to identify strategies to target CD28^null^ T cell (*Figure 1*). Initial studies on small numbers of patients suggested that TNF-α blockade decreases circulating CD28^null^ T-cell number in RA and UA.^25,26^ However, recent studies failed to show consistent depletion of this cell subset in RA patients treated with infliximab or etanercept for 1 year.^27^ Whether TNF-α inhibitors have beneficial effects in patients with coronary atherosclerosis remains to be established.

Statins have also been suggested to reduce CD28^null^ T cells in UA, although the effect was modest (from 3 to 2.3% CD28^null^ T cells, *P*= 0.022).^46^ Moreover, in a small study on patients with myocardial infarction, Rosuvastatin treatment was linked to apoptosis of CD28^null^ T cells analysed *ex vivo*.^47^ We recently demonstrated that Atorvastatin or Rosuvastatin failed to induce apoptosis in CD28^null^ T cells isolated from ACS patients.^14^ Our *in vitro* findings are in line with previous reports that did not identify changes in CD28^null^ T-cell frequency after the acute coronary event in a 2-year follow-up study of ACS patients,^13^ indicating that statins do not have major effects on CD28^null^ T cells.

Protocols that modulate the inflammatory immune response by blocking T-cell co-stimulation are being developed in autoimmunity and other inflammatory disorders. Treatment with a CTLA-4Ig fusion protein (Abatacept) that blocks CD28 ligation on T cells is used in RA. This drug was found to reduce CD8^+^CD28^null^ T cells, but did not influence significantly CD4^+^CD28^null^ T cells in RA patients.^48^ Interestingly, in ACS, we found similar CTLA-4 levels on CD4^+^CD28^null^ and conventional CD4^+^CD28^+^ T lymphocytes, while alternative co-stimulatory receptors OX40 and 4-1BB were markedly up-regulated on CD4^+^CD28^null^ T cells.^10^ This may explain why Abatacept had minor effects on CD4^+^CD28^null^ T cells in RA, and suggest OX40 and 4-1BB blockade as a more rational approach. Importantly, OX40 and 4-1BB are selectively expressed on activated/effector T cells, and are absent from naive/resting lymphocytes. Thus, blockade of OX40 and/or 4-1BB may allow specific modulation of effector T cells that mediate tissue damage, while preserving the ability of naive T lymphocytes to respond to exogenous antigens. Tools to block OX40 and 4-1BB are being developed for RA, multiple sclerosis, inflammatory bowel disease, asthma, transplantation, and graft vs. host disease,^49^ which should facilitate their use in atherosclerosis.

Recently, we have proposed another strategy for targeted modulation of CD28^null^ T cells that exploits molecules that regulate apoptosis. We demonstrated that the pro-apoptotic mitochondrial protein Bim, which has central roles in controlling apoptosis induction, was reduced in CD28^null^ T cells from ACS patients and this associated with apoptosis resistance of these cells.^14^ Moreover, we identified the proteasome, a protein degradation system, as a key molecular switch that controls apoptosis of CD28^null^ T cells by degrading Bim and that, when targeted by proteasome-inhibiting drugs, can restore apoptosis sensitivity of CD28^null^ T cells. Encouragingly, proteasome inhibitors preferentially sensitized CD28^null^ T cells to apoptosis, indicating that the proteasome may be an attractive target to enable selective elimination of CD28^null^ T cells, while sparing conventional CD28^+^ T lymphocytes and avoiding bystander immunosuppression.

Recent studies implicate endogenous microRNAs (miRs) in the regulation of T-cell development, differentiation, and function, and on-going research is trying to harness miRs to target inflammatory responses in atherosclerosis.^50^ Moreover, miRs have important roles in the pathophysiology of cardiovascular diseases. Cardiovascular patients have altered patterns of circulating miRs, and potential diagnostic and therapeutic applications are under investigation.^51^ Of particular relevance to T cells is miR-29 that specifically inhibits IFN-γ production from CD4^+^ T cells by targeting the transcription factors T-bet and Eomes.^52^ MicroRNA-155 has also been implicated in the generation of Th1 cells, as T lymphocytes from miR-155^−/−^ mice showed skewing towards Th2 with predominant production of IL-4 and IL-10 and deficient IFN-γ secretion.^53^ Interestingly, miR-155 was also implicated in Treg survival.^54^ MicroRNA-146a is upregulated in ACS and promotes Th1 differentiation through T-bet induction.^55^ The precise contribution of these miRs to the generation of CD28^null^ T lymphocytes and IFN-γ production is currently unknown. A better characterization of the precise mechanisms that drive the inflammatory/cytotoxic functions and expansion of CD28^null^ T cells may unveil additional strategies to modulate this CD4^+^ T-cell subset in ACS patients.

## Regulatory T cell-based therapies for atherosclerosis

Given their pivotal roles in immune homeostasis and prevention of excessive/harmful immune responses, substantial research efforts are focused on developing Treg-based therapies to reset dysfunctional immune responses in inflammatory diseases. Clinical trials employing Treg are on-going in solid-organ transplantation, type I diabetes, and graft vs. host disease.^56^ These trials primarily use purification of naturally occurring FOXP3^+^ Treg from patients, followed by *in vitro* expansion and reinfusion. Protocols involving *in vivo* manipulation of Treg subsets (expansion of naturally occurring Treg or conversion of antigen-specific conventional T cells into Treg) are also explored (*Figure 1*). Of note, adoptive transfer of Treg in animal models markedly reduced atherosclerosis, suggesting that a similar strategy may be beneficial in patients.^33^ Moreover, several murine studies successfully demonstrate that *in vivo* induction of polyclonal or antigen-specific Treg (e.g. ox-LDL, ApoB100, and HSP60) reduced atherosclerosis development and/or progression.^57^ One of the most promising antigens is apolipoprotein B100 (ApoB100), with several studies showing that ApoB100 peptide-based vaccines inhibited atherosclerosis in mice through Treg induction.^58,59^ Vaccination protocols using ApoB100 are being developed for first-in-human clinical trials.

Immunosuppressive drug treatment has been suggested to affect Treg in atherosclerotic plaques. A randomized control trial on a small group of patients with atherosclerotic carotid artery stenosis found that mycophenolate mofetil, an immunosuppressive drug used to prevent allograft rejection due to its ability to inhibit activated T-cell proliferation, caused not only a reduction in activated (CD3^+^CD69^+^) T cells, but also an increase in CD3^+^Foxp3^+^ in carotid atherosclerotic lesions.^60^

The safety and efficacy of Treg-based therapies in human atherosclerosis has not yet been investigated and important issues remain to be addressed for optimal design of protocols utilizing adoptive transfer of Treg or vaccines that induce Treg *in vivo*. An important challenge for Treg-based immunotherapy remains the stability of Treg during *in vitro* manipulation and following reinfusion in patients with on-going inflammation. Several studies suggest that Treg may lose FOXP3 expression during *in vitro* culture or following reinfusion into hosts, which associates with decreased suppression, and possibly Treg conversion into pathogenic T cells.^32^ Moreover, to efficiently break the chronic inflammation cycle, Treg-based therapies may not be sufficient and may need to be combined with strategies that deplete/modulate pro-inflammatory T cells.

## Future directions

On-going clinical trials testing anti-inflammatory therapies in patients with coronary atherosclerosis target inflammatory cytokines (IL-1β, IL-6, and TNF-α).^4^ Although these trials will bring valuable information on the role of inflammation in atherosclerosis, it is clear that both pro-atherogenic and atheroprotective immune networks operate in this disease. Moreover, considering the complexity and heterogeneity of atherosclerosis in humans, a more targeted immune-modulatory approach, adapted to the predominant pathogenic mechanisms, may be required. Current long-term immunomodulation protocols harbour considerable side effects (infections, immunosuppression, and malignancy). Therefore, careful assessment of benefit/risk profile and development of safer, more targeted immunomodulation therapies are required in atherosclerosis, wherein the inflammatory process is often more discreet than in other chronic inflammatory disorders. Although translation of recent advances on the role of T lymphocytes and other immune cells in atherosclerosis is complex and fraught with challenges, targeted immunomodulation harbours high potential to complement and synergise with drugs/interventions currently used in atherosclerosis patients. Encouragingly, antibodies and other biological or pharmacological reagents that modulate T and B lymphocytes or molecules that regulate their function have been incorporated with promising results in the clinical armamentarium in cancer, autoimmunity, and transplantation, which lends hope to their future applications in atherosclerosis.

## Funding

Work in the Cardiovascular Immunology Laboratory at St George's, University of London is funded by the British Heart Foundation (grant nos PG/10/50/28434, PG/13/24/30115, and PG/14/18/30724) and St George's Hospital Charity, London, UK. Funding to pay the Open Access publication charges for this article was provided by the Charity Open Access Fund provided to St George's, University of London.

**Conflict of interest:** none declared.
